# Proposing a case for integration of screening and management of mental disorders, including substance use disorders with other non-communicable disease care: a prologue to the ICMR-MINDS project in Faridabad district of Haryana

**DOI:** 10.3389/fpubh.2025.1732483

**Published:** 2026-01-09

**Authors:** Yatan Pal Singh Balhara, Siddharth Sarkar, Hitesh Verma, Kuldeep Singh, Gerish Atri, Om Pal Singh Saini, Hanspreet Kang, Ashoo Grover, Pulkit Verma, Neha Dahiya

**Affiliations:** 1 All India Institute of Medical Sciences, New Delhi, India; 2 DGHS Office Punchkula, Punchkula, India; 3 NHM Panchkula, Punchkula, Haryana, India; 4 Indian Council of Medical Research, New Delhi, India

**Keywords:** alcohol, anxiety, comorbidity, depression, health services integration, mental disorders, noncommunicable diseases, primary health care

## Abstract

Mental disorders, including substance use disorders (MSUDs) and other Non-Communicable Diseases (NCDs) together contribute substantially to morbidity, mortality, and economic burden in India. Faridabad district in Haryana is one of the most populous and health system–challenged districts. It exemplifies the urgent need for integrated approaches to address these conditions. Evidence highlights the bidirectional association between MSUDs and NCDs. The Government of India’s National Program for Prevention & Control of Non-Communicable Diseases (NP-NCD), a flagship under the National Health Mission, offers an opportunity to integrate MSUD care into existing service delivery frameworks. Leveraging its established infrastructure, resources, and wide population reach can help improve the care delivery for MSUD. Integrating MSUD into NP-NCD aligns with Sustainable Development Goal 3.4. The Indian Council of Medical Research’s MINDS project in Faridabad district aims to operationalize this integration by implementing and evaluating a scalable service delivery model across 116 health facilities. The initiative aims to integrate the screening, assessment, treatment and referral linkage for MSUD among individuals seeking NCD care. This integrated approach offers a pathway to reduce the treatment gap for MSUD, improve patient outcomes, and inform adoption of evidence-based, sustainable health strategies at a larger scale in the state of Haryana.

## Introduction

### Faridabad district: overview of health system

Faridabad is the largest and most populous (population more than 2.4 million) district in the state of Haryana in Northern India. This district has a combination of rural and urban areas. The public health in the district is delivered through a tiered health system. The primary level tier includes Aayushman Aarogya Mandir- Sub centers (AAM-SCs), Aayushman Aarogya Mandir- Primary Health Centres (AAM-PHCs), and Community Health Centres (CHCs). AAM- SCs are the first level of contact and are staffed by staffed by frontline health workers including Community Heath Officers (CHOs) and Accredited Social Health Activist (ASHA). AAM- PHCs provide outpatient care, essential drugs, minor procedures, and routine health monitoring under a Medical Officer. CHCs serve as referral hubs for AAM-SCs and AAM- PHCs. They offer basic specialist care, in-patient beds, diagnostics, and emergency services. The secondary tier is housed in the District Hospitals and sub-district hospitals, which provide surgery, obstetric care, pediatrics, general medicine, trauma care, blood storage, and advanced diagnostics. From administrative perspective these health facilities are organized in blocks. There are a total of four blocks in the rural areas. The urban areas facilities constitute the fifth set of health care facilities. In addition, the Comprehensive Rural Health Services Project (CRHSP) located at Ballabgarh, provides preventive, health-promotion, and curative services to its surrounding population.

Despite its proximity to the national capital (New Delhi), Faridabad continues to exhibit wide disparities in health outcomes, particularly concerning chronic disease care and mental health service availability. This reflects the systemic gaps between health policy design and ground-level implementation. An evaluation of the health system performance at district level in India reported Faridabad to be in the category of the poorest performing districts in Haryana (1). It received a score of 0.49 out of 1 on the Health System Performance Index (HSPI) and ranked among the lowest districts within the state. The district had among the lowest number of core public health workers per 10,000 population (1.35) compared to other districts. There were shortcomings in health facility readiness including availability of essential equipment, drugs, supplies, and functional infrastructure in public facilities. There was poor quality, completeness, or usability of the health information system. There was lower utilization of services or inefficiencies in the service delivery process.

A more recent assessment of the mental health related stigma, service provision and utilization in the district reported poor awareness and knowledge about mental illnesses (stigma in both general public and health workers), help-seeking behaviors being driven by local contextual factors (myths, beliefs, and practices), poor availability of mental health services at primary healthcare centers, and inadequate mental health facilities at secondary or tertiary levels, dearth of trained mental health professionals and psychotropic medicines, and inadequate finances, logistics and infrastructure (2).

Another study assessing the health services in the rural areas of the district found that 27.3 and 9.1% of the respondents believed the overall health of people in their village was average and poor, respectively. In addition, the majority of villages reported addiction to alcohol and tobacco (54.5%) a major health issue (3). Concerns have also been expressed with regards to poor utilization of digital data entry platforms in the district.

### Growing burden of non-communicable diseases in India and shift in priorities

Non-Communicable Diseases (NCDs) are the leading cause of morbidity and mortality in India. The NCDs accounted for more than 60% of all deaths in the country (4). In 2017, approximately 4.7 million deaths occurred due to NCDs that comprised 49% of all-cause mortality. Of these deaths cardiovascular diseases (23%), chronic respiratory diseases (9%), cancer (6%) and diabetes (2.4%) were the major causes. In addition, the Disability Adjusted Life Years (DALYs) for NCDs accounted for 47% of all-cause DALYs (5). The losses due to NCDs are also projected to increase over the years. A study among adolescents in the rural areas of the Faridabad district found the prevalence of hypertension among adolescents to be close to the prevalence level of hypertension among adults. This signaled rising levels of hypertension in adulthood and thus potential increase in cardiovascular morbidities in the district (6).

There has been a shift in the priorities of health policy in the country over the past decade. The Government of India launched the National Program for Cancer, Diabetes, Cardiovascular Diseases and Stroke (NPCDCS) in 2011 across 100 districts in 21 states. The program aimed to prevent and control NCDs. The various strategies to achieve the same include behavioral change communication, community participation, opportunistic screening of NCDs and provision of NCD services through public health facilities (7). The National Health Mission (NHM) took over the program in 2013–2014. It was done with an aim to optimize resources, provide seamless services to patients, and to ensure long term sustainability of the program. The Government of India has identified additional priority NCD conditions beyond common NCDs, expanding the program’s scope to include Chronic Obstructive Pulmonary Disease (COPD), Asthma, Chronic Kidney Disease (CKD), Non-Alcoholic Fatty Liver Disease (NAFLD), and the Pradhan Mantri National Dialysis Program (PMNDP). This is due to the growing burden of NCDs and associated morbidities and mortalities. The National Program for Prevention & Control of Non-Communicable Diseases (NP-NCD) has since replaced the NPCDCS.

A report by the Ministry of Health and Family Welfare (MoHFW) indicates that the NP-NCD has achieved significant coverage expansion, with total enrolment (30 years+) through NCD application crossing 400 million, and 390 million screened by the end of 2024. However, mental health integration remains limited, underscoring the urgent need for systemic inclusion.

We hereby present a case for integration of services for mental disorders, including substance use disorders (MSUDs) in the NCD care being offered as part of the NP-NCD in Faridabad District of Haryana. This proposition has its origin in a project funded by the Indian Council of Medical Research titled “Implementation Research Study on Integration of Screening and Management of Mental and Substance Use Disorders with Other Non-Communicable Diseases (ICMR-MINDS).” ICMR- MINDS is one of the National Health Research Priority projects of ICMR (Indian Council of Medical Research) (8). This integrated model in Faridabad district is expected to generate real-world evidence for scaling up mental health integration efforts, offering a replicable blueprint for health systems in other districts in the state and similar contexts elsewhere. While the need to have an integrated approach in context of MSUD and other NCDs has been expressed earlier, the real world implementation of the same remains limited (9).

The aim of this article is to present a clear and evidence-based case for integrating the screening and management of MSUDs into the ongoing NCD care in Faridabad district of Haryana. The article draws on the epidemiological evidence, documented health-system gaps, and the established bidirectional relationship between MSUDs and other NCDs. It outlines the rationale for strengthening public-health delivery through an integrated approach in Faridabad district of Haryana.

## The bidirectional association between MSUDs and NCDs

The MSUDs and NCDs have an intricate bidirectional association. The rate of co-occurrence of MSUDs and NCDs is much higher than what can be explained by chance. People with diabetes are more likely to develop depression, and depression is a risk factor for diabetes. Depression and anxiety predict the development of cardiovascular disease (CVD) and worsen its prognosis. Anxiety and depression are more common among people with respiratory diseases. A large proportion of people with cancer have a common mental disorder such as anxiety or depression. Moreover, a dose–response association was seen between an increasing number of mental disorders and CVD (10). People with severe mental disorders die 10–20 years or even earlier than the general population, primarily due to preventable NCDs such as heart disease and metabolic syndrome (11). In addition, the co-occurrence of mental disorders and substance use disorders has been reported from India (12, 13).

The association between the MSUD and NCDs has been reported in the Faridabad district. In a community-based case–control study in 28 villages of Ballabgarh block of Faridabad district common mental disorders were found to be more among patients with diabetes mellitus as compared to the controls (adjusted odds ratio—3.2, 95% confidence interval: 1.9–5.2) (14).

The causal association between MSUDs and NCDs can be attributed to a complex interplay of individual and environmental factors (15). The risk factors for NCDs tend to cluster together in people with MSUDs. People with mental disorders have a higher rate of use of nicotine and alcohol. Both are the leading risk factors for NCDs. Tobacco and alcohol were documented as risk factors for NCDs in the National noncommunicable disease monitoring survey (NNMS) in India (16). The age-adjusted prevalence forever use of tobacco and alcohol was found to be 34.2 and 23.4%, respectively among young men aged 18–24 years in the rural areas in Faridabad district (17). In addition, lower levels of physical activity among the persons with mental disorders can also contribute to an increased risk of various NCDs. Also, evidence has accumulated that suggests that the risk factors for NCDs viz. lifestyle risk factors, poor diet, physical inactivity, smoking and alcohol use, also contribute to the onset and trajectory of the mental disorders (11).

## Economic and societal impact

The economic and social impacts of NCDs and MSUD in India is significant. Both NCDs and MSUD put a significant financial burden by means of out-of-pocket health expenditure, loss of household income and productivity losses. These can exacerbate poverty (10). Moreover, the economic implications of NCDs threaten the achievement of various Sustainable Development Goals (SDGs). These include those related to poverty reduction, inequality reduction, hunger alleviation, access to quality education, and gender equality (11).

According to the World Bank’s 2024 analysis, approximately 55% of healthcare spending in India remains out-of-pocket. When MSUD and other NCD coexist, this financial strain multiplies, often resulting in medical debt, loss of employment, and significant economic disadvantage. Previous reports estimated that India is expected to incur productivity losses exceeding USD 4.5 trillion between 2012 and 2030 due to NCDs and mental health conditions combined (18).

## Movement toward achieving sustainable development goals

The target 3.4 for the Sustainable Development Goals (SDGs) is to reduce by one third premature mortality from non-communicable diseases through prevention and treatment and promote mental health and well-being. The World Health Organization’s Global Mental Health Action Plan (2023–2030) emphasizes that mental health integration into universal health coverage (UHC) is essential for achieving SDG 3.4. Integration of services (promotion, prevention, treatment, and rehabilitation) for MSUDs and other NCDs offers a viable option to achieve this target. This integration also directly contributes to SDG 3.8 (Universal Health Coverage) and SDG 10 (Reduced Inequalities) by ensuring equitable access to mental health services within existing NCD systems.

## Possibility of opportunistic screening

The NP-NCD offers an option for opportunistic screening and intervention for MSUD. This leveraging of an existing mechanism, whereby the community has been put in contact with the public health care system, helps address multiple challenges for the mental health care delivery including inadequate infrastructure, a shortage of trained personnel, insufficient funding, and a lack of public awareness about mental health in the country. This opportunistic screening can bridge the treatment gap for MSUDs by reaching individuals who may not otherwise seek mental health care due to stigma or unawareness. Integration within NCD services thus offers both efficiency and equity benefits. It has been proposed that the National Mental Health Program (NMHP) can be strengthened by the learnings of the NP- NCD and the approach used in the NP-NCD can help improve the outcomes of NMHP (19).

## Leveraging a flagship national program

The NP- NCD is one of the flagship programs of India under the National Health Mission (NHM). The flagship status of the program offers support to the availability of resources and sustenance of the program over the years. Over the years the budget allocated for the NMHP has been under 1% of the total health budget of the country (20). This has been a major limiting factor to the scaling up of MSUD services in the country. The analysis of central and state budgets published in 2019 reported that the states spent about 80% of the health budget for NCDs while the central government spent 65% of the total health budget on NCDs (21). The budget approved under the State Program Implementation Plans (PIPs). For the NP- NCD for the financial year 2024–25 is much higher at ₹1,683.97 crores. In addition, the NP- NCD has a much wider reach as 770 District NCD Clinics have been established under the program. This contrasts with the NMHP which has not yet reached all the districts of the country despite a pick-up in pace over the last decade (22). The NP- NCD was rolled out in the year 2010, as compared to the NMHP that was initiated way back in the 1980s.

## Mental disorders, including substance use disorders a type of NCD

**Figure 1:**
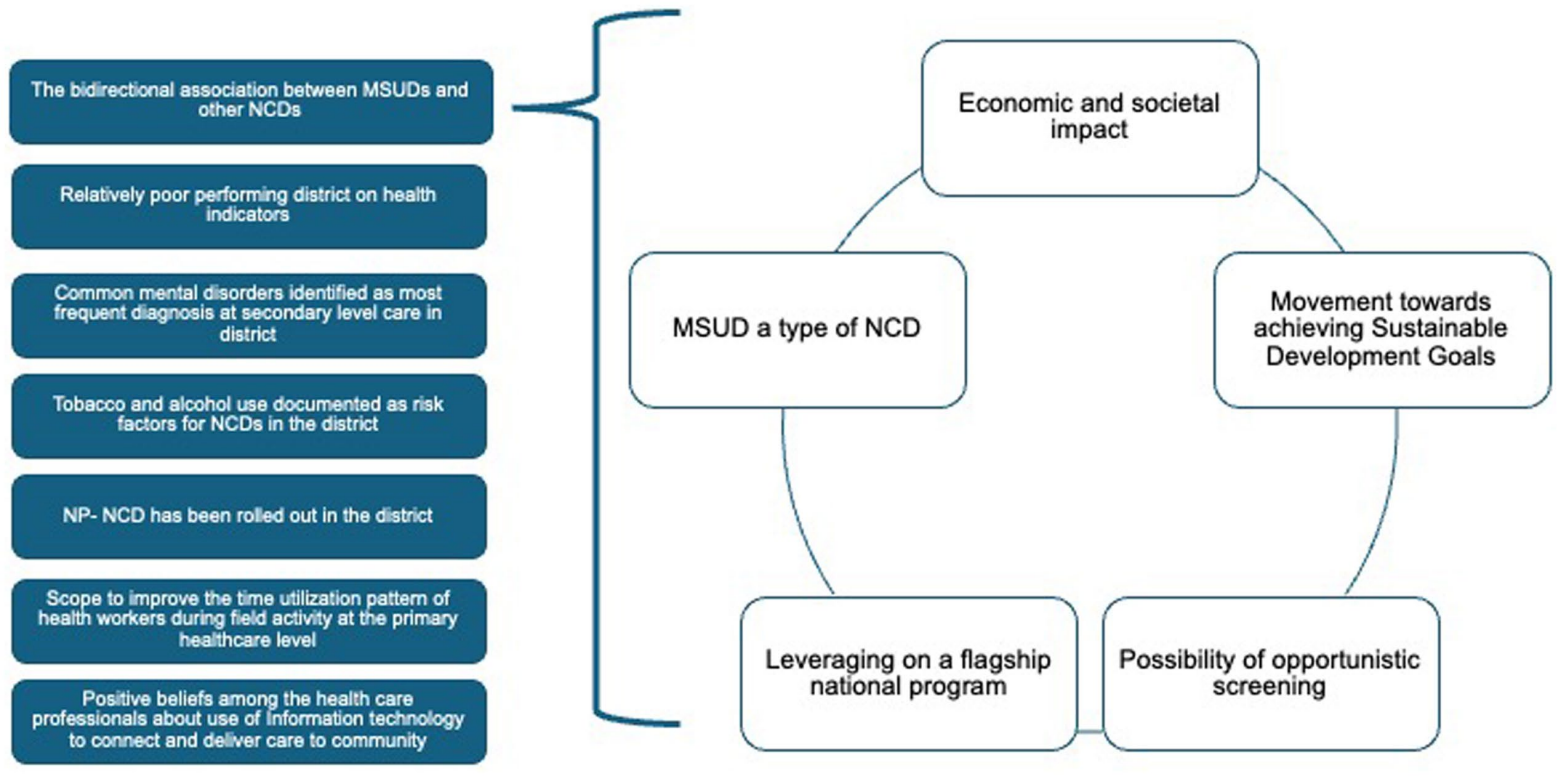
Rationale for integration of screening and management of mental disorders, including substance use disorders (MSUD) within other non-communicable diseases (NCDs) care in Faridabad district of Haryana state.

Conceptually and epidemiologically, MSUDs share multiple characteristics with other NCDs including chronicity, multifactorial causation, long latency, and need for continuing management. It can be argued that MSUD belongs to the broader category of NCDs. This is not a new proposition as arguments have been made in the past for the same (23). The WHO and the United Nations Inter-Agency Task Force on NCDs (IATF) reaffirmed that mental health conditions should be integrated within the global NCD response to address the double burden faced by low- and middle-income countries. Therefore, there is no rationale for leaving MSUD out of the purview of the flagship national program in the form of NP-NCD. While there is a separate National Mental Health Program (NMHP) in the country that is focused on MSUD, the limitations of this program have been documented (19). Adopting such a unified approach can improve cost-effectiveness, enhance resource utilization, and strengthen health equity by ensuring that MSUD care is seen as an integral component of chronic disease care (Figure 1).

## ICMR- MINDS in Faridabad district of Haryana: an attempt to strengthen the integrated health care delivery for MSUD

The Faridabad district was identified in consultation with the state health authorities. Since the study included extensive field work, the proximity of the Faridabad district to the host institution of the project investigators guided the choice. The district comprises both urban and rural areas. Given the systemic limitations of the district, there is an opportunity to design and implement the project in a challenging setting. Demonstrating the implementation of the project in such a setting shall help build a strong case for scaling up the model at the level of the whole state.

**Figure 2:**
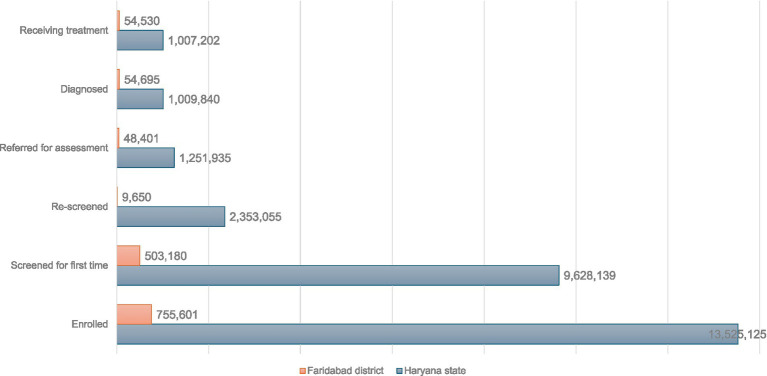
Cases enrolled, screened, rescreened, referred for assessment, diagnosed and treated for both Haryana state and Faridabad district as part of NP-NCD as of third week of October 2025.

The NP- NCD is operational in the Haryana state including the Faridabad district (24). The details of the cases enrolled, screened, rescreened, referred for assessment, diagnosed and treated for Haryana state and Faridabad district have been presented in Figure 2.

There are multiple district-level system features supporting integration of MSUD care into existing care for other NCDs in Faridabad district. These have been summarized in Table 1.

**Table 1:** District-level system features supporting integration of MSUD care into existing care for other NCDs in Faridabad district.

NP- NCD is operational in the Faridabad district of Haryana state Common mental disorders identified as most frequent diagnosis at secondary level care in the district Tobacco and alcohol use documented as risk factors for NCDs in the district Limited qualified mental health professionals in the public health system in the district Pre-existing screening questions for tobacco and alcohol use Inclusion of two question screener for depression (PHQ-2) Sensitization of the health care workers about prioritizing the care delivery for NCDs Opportunity to take a whole of system approach by developing services shall be developed for all levels of health care delivery Emphasis on the Comprehensive Primary Health Care (CPHC) Reaching out the community by piggybacking on the population-based screening Established channels for NCD care across different levels of health care delivery Population in the Faridabad district receptive to public health advice (learning from COVID-19 pandemic) Scope to improve the time utilization pattern of health workers during field activity at the primary healthcare level Positive beliefs among the health care professionals about the use of Information technology to connect and deliver care to the community Opportunity to design and implement the project in a challenging setting

Common mental disorders consisting of mood disorders and neurotic stress -related were two of the most commonly diagnosed disorders among reported psychiatric morbidity at the secondary level public health facility in the Faridabad district is a previous study (25). However, a scarcity of psychiatric manpower and facilities in the district with a distinct urban skew has been reported for the Faridabad district (26). This is in keeping with a more generic limited focus on MSUD training of health professionals in the ow and middle income countries (LMIC) globally (27). Common mental and substance use disorders are the focus of the ICMR- MINDS project. Also, by including all cadres of health care professionals to provide the MSUD services the project would be able to address the scarcity of qualified mental health professionals in the district.

The integration of MSUD care in the NCD care shall help to achieve the objectives of the NMHP and help narrow the treatment gap for MSUD in the Faridabad district. A previous study reported that there was a scope to improve the time utilization pattern of health workers during field activity at the primary healthcare level in the Faridabad district (28). This can help with more efficient utilization of the existing human resources to strengthen health care delivery in the district.

ICMR- MINDS is one of the National Health Research Priority projects of ICMR (Indian Council of Medical Research). The primary objective of the study is to develop and implement a service delivery model that would result in high coverage of screening, management, and linkage to care for common MSUDs among individuals seeking treatment for NCDs at public health facilities. The detailed study protocol has been published (8).

The initiative also emphasizes a whole-of-system approach as it is being implemented across all levels of public health facilities in Faridabad district in Haryana. We intend to roll out the initiative across a total of 116 public health facilities. These included Ayushman Arogya Mandir Sub Centres (AAM- SC), Ayushman Arogya Mandir Primary Health Centres (AAM- PHC), Community Health Centre (CHC); Sub Divisional Hospital, and District Hospital across rural, and urban areas in the district.

A previous study assessed the beliefs of frontline health workers working in the rural areas of Faridabad district about the use of Information technology to connect and deliver care to the community. Various positive beliefs were identified. These are related to improvement in work efficiency and social status, less paperwork, timely report generation, and better learning. The negative beliefs were related to an increase in working hours, close monitoring, and feeling over-burdened (29). This is of relevance in context of ICMR- MINDS project as it involves use of mobile based Clinical Decision Support System (CDSS) to deliver integrated care for MSUD. Use of such digital tools remains limited in LMICs (30). Need for integration and adequate utilization of web-based health information portals to ensure provision of health services at the primary health care level in the district has been identified previously (31).

The population in the Faridabad district has been found to be receptive to public health advice. The rural community of Ballabgarh block in the district showed positive practices with respect to prevention of COVID-19 in a previous study (32). Public engagement and awareness generation about MSUD is one of the key interventions for the ICMR- MINDS project. A good uptake of this intervention is expected in the district. Previous efforts aimed at strengthening public health care delivery in the Faridabad district have also yielded desirable results. Institutional delivery increased by almost 2.7 times after launch of a targeted program in the district (33).

We foresee some potential health system and community level challenges to the implantation of this project in Faridabad district. These include the workforce deficit, facility readiness gaps, supply threats, low awareness, stigma, disclosure risk, adoption risk for project interventions (innovations), urban skew, rural access limit among others. The proposed interventions (innovations) of the ICMR- MINDS project in form of tools for integrated screening and assessment for MSUD for all levels of healthcare personnel; the workflows and pathways that integrate MSUD interventions within NCD care; digital platform and smartphone based CDSS; trainings of the healthcare professionals; and content aimed at patient education and engagement to raise awareness and promote shared decision-making shall aim to mitigate some of these challenges. In addition, active stakeholder engagement and co- creation of the implementation strategies as part of the implementation model shall be the key.

This initiative in Faridabad district of Haryana aims to achieve comprehensive integration of MSUD services within the existing NCD care framework in the public health system. A key expected outcome is screening, linkage to care, and management of MSUD among patients seeking NCD care. This includes improved detection rates, timely referrals, and effective treatment initiation for MSUDs at all levels of healthcare. The initiative also anticipates enhanced healthcare provider capacity across the whole of the public health system in the district. Ultimately, the initiative seeks to generate scalable, evidence-based strategies for statewide adoption of integrated mental health and NCD care in the state’s public health infrastructure.
