# Supplementary figures and images for: Proposing a case for integration of screening and management of mental disorders, including substance use disorders with other non-communicable disease care: a prologue to the ICMR-MINDS project in Faridabad district of Haryana
Source: Front Public Health. 2026 Jan 9;13:1732483. doi: 10.3389/fpubh.2025.1732483 (PMC12827729; doi:10.3389/fpubh.2025.1732483)

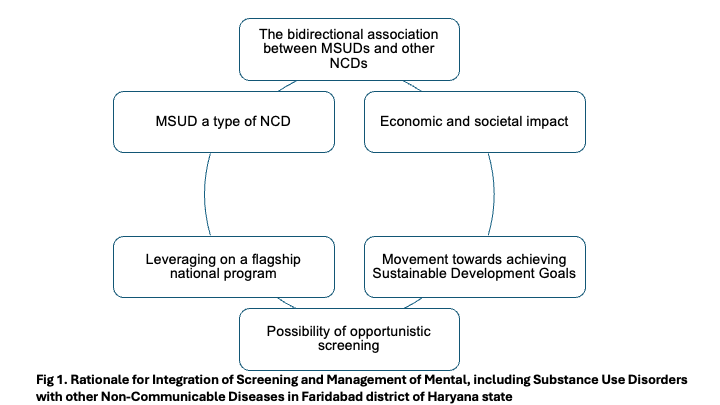

Supplement: Supplementary file 1 [file Image_1.png]
